# Treatment of malignant tumors of the skull base with multi-session radiosurgery

**DOI:** 10.1186/1756-8722-2-16

**Published:** 2009-04-02

**Authors:** Nicholas D Coppa, Daniel MS Raper, Ying Zhang, Brian T Collins, K William Harter, Gregory J Gagnon, Sean P Collins, Walter C Jean

**Affiliations:** 1 Department of Neurosurgery, Georgetown University Hospital, Washington, DC, USA; 2 Department of Radiation Oncology, Georgetown University Hospital, Washington, DC, USA; 3 Biostatistics Unit, Lombardi Comprehensive Cancer Center, Georgetown University Medical Center, Washington, DC, USA; 4 Faculty of Medicine, University of Sydney, Sydney, Australia

## Abstract

**Objective:**

Malignant tumors that involve the skull base pose significant challenges to the clinician because of the proximity of critical neurovascular structures and limited effectiveness of surgical resection without major morbidity. The purpose of this study was to evaluate the efficacy and safety of multi-session radiosurgery in patients with malignancies of the skull base.

**Methods:**

Clinical and radiographic data for 37 patients treated with image-guided, multi-session radiosurgery between January 2002 and December 2007 were reviewed retrospectively. Lesions were classified according to involvement with the bones of the base of the skull and proximity to the cranial nerves.

**Results:**

Our cohort consisted of 37 patients. Six patients with follow-up periods less than four weeks were eliminated from statistical consideration, thus leaving the data from 31 patients to be analyzed. The median follow-up was 37 weeks. Ten patients (32%) were alive at the end of the follow-up period. At last follow-up, or the time of death from systemic disease, tumor regression or stable local disease was observed in 23 lesions, representing an overall tumor control rate of 74%. For the remainder of lesions, the median time to progression was 24 weeks. The median progression-free survival was 230 weeks. The median overall survival was 39 weeks. In the absence of tumor progression, there were no cranial nerve, brainstem or vascular complications referable specifically to CyberKnife^® ^radiosurgery.

**Conclusion:**

Our experience suggests that multi-session radiosurgery for the treatment of malignant skull base tumors is comparable to other radiosurgical techniques in progression-free survival, local tumor control, and adverse effects.

## Introduction

A variety of malignant tumors can involve the skull base. These tumors may originate from various tissues of the skull base, or invade into the region as extensions of head and neck cancers [1,2]. The skull base is also a common site of metastasis from distant tumors [3,4]. Patients with skull base malignancies suffer greatly [5]. Common clinical presentations include pain and cranial nerve deficits, such as visual disturbances, facial paresis and swallowing difficulties [3]. Treatment of these tumors presents formidable challenges to the clinician. In addition to neurological factors, such as the close proximity of critical neurovascular structures, oncological factors play a key role. Metastatic skull base tumors are often late complications of systemic cancers, and the advanced systemic tumor burden, poor overall clinical condition and the morbidities from prior interventions, all make treatment difficult [6,7].

Historically, malignant skull base tumors were deemed inoperable and the overall prognosis was poor, especially for those presenting with cranial nerve deficits [8,9]. Surgical resection was frequently incomplete and limited by high mortality, risk of severe neurological morbidity and frequent recurrences [10-13]. Important technical advancements such as improved understanding of the microanatomy of the area, higher-resolution diagnostic imaging, safer operative strategies, and multidisciplinary collaboration have evolved over the past three decades, making surgical treatment safer [14,15]. Surgical resection or debulking is currently considered a critical component of their management [16,17]. But, even though some authors regard surgery as the "gold standard" treatment, the limitations of brainstem and cranial nerve morbidities continue to make curative resections a rarity [18-20].

There is an important role for radiation therapy in the management of skull base malignancies, both as primary treatment as well as adjuvant treatment, after surgical resection [21-26]. However, as with surgery for these tumors, the limitations of this therapy are readily apparent. External beam radiation therapy alone results in poor local control and overall survival due to factors such as large tumor volume, limitations of radiation dose, and the intrinsic "radio-resistance" of certain tumors [27,28]. Single-session radiosurgery has been employed in the treatment of chordomas and malignant tumors at the cranial base [3,29-34]. However, given the close proximity of these lesions to critical neurovascular structures, methods to minimize radiation-induced toxicities should be considered. [35-45]. More recently, "hypofractionated" or staged radiosurgery has provided an attractive alternative. This therapy has been successfully utilized in the treatment of tumors in which preservation of surrounding structures is particularly vital, such as those near the optic nerve and optic chiasm, as well as for various lesions at the skull base [46-49]. The hiatus between treatment sessions theoretically provides time for normal tissue repair, and the resultant lower radiation risk to the normal structures permits more effective treatment of the target lesion [50]. This therapy may be particularly useful for patients with skull base malignancies, for whom the essential goal of treatment is for palliation rather than cure [31].

The CyberKnife^® ^is an image-guided, frameless radiosurgical system that uses inverse planning for the delivery of radiation to a defined target volume [51]. Non-isocentric radiation delivery permits simultaneous treatment of multiple lesions, and the frameless configuration allows for staged treatment. It has been successfully utilized to treat various skull base lesions including chordomas and plasmacytomas among many others [47,49]. We utilized the CyberKnife^® ^to treat skull base malignancies, believing that it is useful for managing these relatively rare but highly challenging tumors. In this retrospective study, we evaluated the efficacy and safety of staged stereotactic radiosurgery for treatment of malignant skull base tumors, either as a primary treatment modality or as an adjunct to surgery and conventional external beam radiotherapy.

## Patients and methods

### Patient Population

We performed a retrospective review of 464 patients with intracranial tumors who were treated with CyberKnife^® ^stereotactic radiosurgery (CKS) at Georgetown University Hospital between January 2002 and December 2007. One hundred forty-five patients were classified as having tumors of the skull base, of which 108 were benign. Thirty-seven patients had 37 lesions that were classified as malignant skull base tumors. Six patients who had follow-up periods less than or equal to four weeks were eliminated from statistical consideration, thus leaving 31 patients for analysis.

For the purposes of this study, skull base lesions were defined as those that involved the osseous structures of the base of the skull, in close proximity to the critical neurovascular structures of the region. All the tumors included in this study either completely encircled, partially circumscribed, or directly contacted the brainstem, optic chiasm, or cranial nerves with meaningful remaining function. Primary brain tumors were excluded, unless they had the potential to metastasize and were thus considered malignant. An example of such a tumor is a hemagiopericytoma. Malignant orbital, sinus and head-and-neck tumors were included in this study only if there was intracranial extension.

This malignant skull base tumor group consisted of 21 men and 10 women, with a median age of 57 (range: 11 – 81) (Table 1). The histopathology of all tumors was either known from prior microsurgical resection, biopsy, or was presumed based on the intracranial extension of known head and neck cancers.

**Table 1:** Patient characteristics

	**Study Group**
Number of patients	31
Number of lesions	31
Gender	
Male	21
Female	10
Age	
Min	11
Max	81
Median	53
Mean	57

### Radiosurgical Treatment Planning and Delivery

A multidisciplinary meeting of specialists that included neurosurgeons, otolaryngologists, radiation oncologists, medical oncologists, and neuroradiologists evaluated all patients. A collective decision to treat with radiosurgery was made for each individual patient. Radiosurgery was only offered to patients for whom conventional microsurgical resection was contraindicated because of high neurological risk, overwhelming medical comorbidities, poor prognosis with limited survival, or recurrent disease in the presence of prior microsurgical resection, chemotherapy and radiation therapy.

The CyberKnife^® ^radiosurgical system was used to administer cranial radiosurgery in every case. The technical aspects of CKS for cranial tumors have been described in detail [46,50]. Briefly, the patient's head was immobilized by a malleable thermoplastic mask during the acquisition of a thin-sliced (1.25 mm) high-resolution computed tomography scan, which was used for treatment planning. The use of a contrast-enhanced MRI fused to the treatment planning CT scan was at the discretion of the treating physicians. This decision was influenced by various factors, such as previous radiation to the area, performance status, treatment intent and extent of contact and compression of critical neurological structures. The target volumes and critical structures were then delineated by the treating neurosurgeon. An inverse planning method with non-isocenteric technique was used for all cases, with specific dose constraints on critical structures such as the optic chiasm and brainstem. The planning software calculated the optimal solution for treatment, and the dose-volume histogram of each plan was evaluated until an acceptable plan was found. The treating neurosurgeon and radiation oncologist, who have a shared responsibility for all aspects of the treatment planning and procedure, determined the minimal tumor margin dose of the target volume, the treatment isodose and the number of treatment sessions into which the total dose was to be divided. This decision was influenced by various factors, such as previous radiation to the area, tumor volume, and extent of contact and compression of critical neurological structures. In most cases, the treatment dose was prescribed to the isodose surface that encompassed the margin of the tumor.

The delivery of radiosurgery by the CyberKnife^® ^was guided by real-time imaging. Using computed tomography planning, target volume locations were related to radiographic landmarks of the cranium. With the assumption that the target position is fixed within the cranium, cranial tracking allowed for anatomy based tracking relatively independent of patient's daily setup. Position verification was validated several times per minute during treatment using paired, orthogonal, x-ray images.

### Caclulation of Radiosurgical Treatment Planning Parameters

The homogeneity index and new conformity index were calculated for each treatment plan. The homogeneity index (HI) describes the uniformity of dose within a treated target volume, and is directly calculated from the prescription isodose line chosen to cover the margin of the tumor. It is calculated by the following equation:



The new conformity index (NCI) as formulated by Paddick, and modified by Nakamura, describes the degree to which the prescribed isodose volume conforms to the shape and size of the target volume [52,53]. It also takes into account avoidance of surrounding normal tissue. It is calculated by the following equation:



### Clinical Assessment and Follow-Up

Post-radiosurgical follow-up was typically performed in a multidisciplinary clinic of the treating neurosurgeon and radiation oncologist beginning one month after the conclusion of radiosurgery. Patients were subsequently followed in three-month intervals. During each follow-up visit, a clinical evaluation and physical examination were performed as well as a review of pertinent radiographic imaging. If a patient experienced deterioration in their clinical condition at any point during the follow-up period, an immediate evaluation was performed. The progress of all patients was discussed periodically at a multidisciplinary tumor conference of various specialists, ensuring precise interpretation of the available data. We analyzed tumor response, clinical outcome, treatment-related complications and survival during the follow-up period.

## Results

### Patient and tumor characteristics

The characteristics of the study group including the distribution of gender, age, tumor histology and location are detailed below and summarized in Tables 1 and 2. The most frequent tumors in this series were squamous cell carcinoma (6 lesions), adenoid cystic carcinoma (5 lesions), rhabdomyosarcoma (2 lesions) and metastases of melanoma and renal cell carcinomas (3 lesions each). The median tumor volume was 18.3 cc (range: 3.2 – 206.5 cc).

**Table 2:** Skull base tumor characteristics

	**Study Group**
Volume (cc)	
Min	3.2
Max	206.5
Mean	41.6
Median	18.3

Histology	
Adenoid cystic carcinoma	5
Breast cancer	1
Chondrosarcoma	1
Ewing sarcoma	2
Hemangiopericytoma	1
Hepatocellular carcinoma	1
Leiomyosarcoma	1
Melanoma	3
Papillary thyroid carcinoma	1
Parotid adenocarcinoma	2
Renal cell carcinoma	3
Rhabdomyosarcoma	2
Spindle cell carcinoma	1
Squamous cell carcinoma	6
Transitional cell carcinoma	1

Location	
Cavernous sinus	8
Cribriform plate	1
CP angle/IAC	2
Ethmoid	1
Foramen magnum	1
Foramen ovale	1
Infratemporal fossa	3
Jugular foramen	1
Middle fossa	2
Parasellar	1
Orbit	7
Petroclival	3

Goal of CyberKnife treatment	
Primary treatment for local disease (%)	18 (58)
Secondary treatment (%)	13 (42)
Previous treatment	
Previous craniofacial surgery	6
Previous external beam radiation	4
Previous stereotactic radiosurgery	1
Previous biopsy only (%)	4

Tumors varied in their skull base location, as illustrated in Table 2. A number of lesions, however, spanned multiple anatomical locations. CKS was the primary treatment to the malignant skull base tumor in 18 patients (58%). Of the 13 patients with previous treatment to the tumor involved in this study, 6 (46%) had previous craniofacial surgery, 4 (30%) had previous external beam radiation, and 1 (7%) had previous stereotactic radiotherapy. Four patients (13% of the entire series) had undergone biopsy only.

### Radiosurgical treatment

The specific dose and fractionation scheme for the tumors in this series was influenced by various factors, including previous radiation to the area, tumor volume, and extent of contact and compression of critical neurological structures. Details of the radiosurgical treatments are found in Table 3. A median treatment dose of 2500 cGy was delivered to the margins of the tumors in this study (range: 1260 – 3500 cGy). Radiosurgery was delivered during a median number of 5 sessions (range: 2 – 7) on a median isodose line of 75% (range: 68 – 88%) as defined at the margin of the treated tumor. The median homogeneity index (HI), a measure of dose homogeneity to the tumor, was 1.32 (range: 1.11 – 2.44). For the lesions where it was available (28 lesions), the median new conformity index (NCI) was 1.60 (range: 1.29 – 2.59).

**Table 3:** Radiosurgery treatment plan

	**Study Group**
Dose (cGy)	
Min	1260
Max	3500
Mean	2449
Median	2500

Treatment Stages	
Min	2
Max	7
Mean	4.45
Median	5

Homogeneity Index	
Min	1.14
Max	2.44
Mean	1.34
Median	1.32

New Conformality Index	
Min	1.29
Max	2.59
Mean	1.70
Median	1.60

Isodose Line (%)	
Min	68
Max	88
Mean	77
Median	75

### Tumor Control

**Figure 1:**
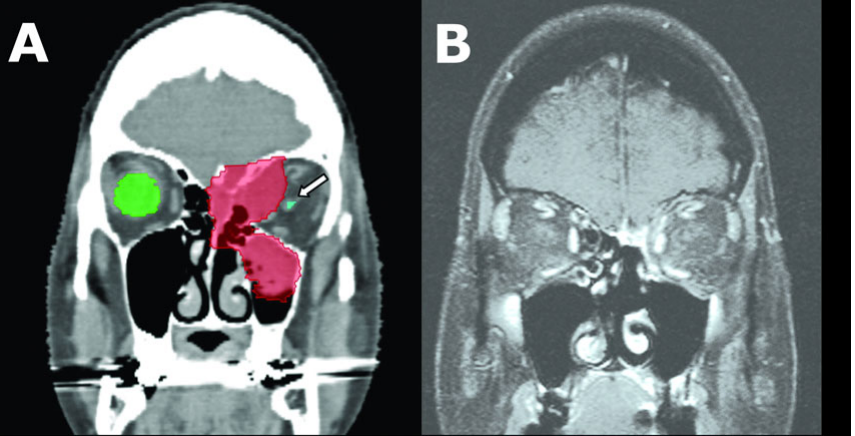
**57-year-old woman with squamous cell carcinoma of the left ethmoid sinus, orbit and anterior skull base**. Prior to consideration of radiosurgery, the original treatment plan was craniofacial resection with left orbital exenteration. She was treated with 3000 cGy in 5 stages. (A) Coronal CT with contrast prior to radiosurgery with treatment-planning contour. The tumor is shaded in red. Note proximity of left optic nerve. *White arrow: optic nerve*. (B) Coronal MRI with contrast 13 months after radiosurgery showing dramatic response. Currently, the patient continues to have normal binocular vision nearly 4 years after treatment.

**Figure 2:**
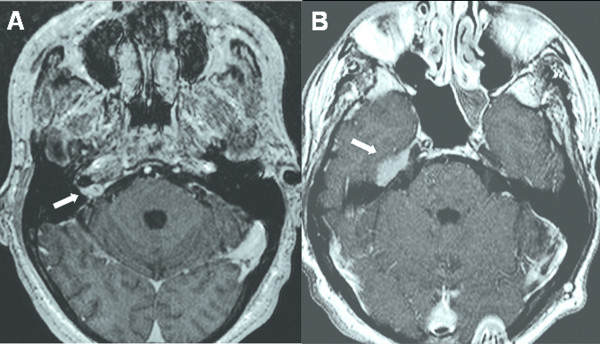
**50-year-old man with biopsy-proven renal cell carcinoma to the right internal acoustic meatus (IAM)**. He was treated with 2500 cGy in 5 stages. (A) Axial MRI with contrast prior to radiosurgery showing the tumor at the IAM. *White arrow: tumor*. (B) Axial MRI with contrast 5 months after radiosurgery showing extension of disease cephalad. This area was treated with an additional 2400 cGy in 3 stages. *White arrow: tumor extension*.

The median follow-up was 37 weeks (range: 6 – 238 weeks) (Tables 4 &5). At last follow-up, or at the time of death from systemic disease, 5 tumors (16%) had regressed, and 18 (58%) exhibited stable local disease (Figure 1 and Table 4). Eight lesions (26%) progressed locally despite treatment (Figure 2). The overall tumor control rate in these 31 patients was 74%.

**Table 4:** Treatment outcomes after CyberKnife radiosurgery

	**Study Group**
Follow-up (weeks)	
Min	6
Max	238
Mean	54
Median	37
Survival at last follow-up (%)	10 (32)
Time to Death	
Min	6
Max	142
Mean	32
Median	25
Local disease outcome	
Disease regression (%)	5 (16)
Stable disease (%)	18 (58)
Disease progression (%)	8 (26)
Death due to treated disease (%)	0 (0)
Time to local progression (weeks)	
Min	5
Max	230
Mean	47
Median	24

**Table 5:** Treatment outcomes after CyberKnife radiosurgery

**Patient**	**Histology**	**Prior Surgery**	**Prior Radiation**	**Local Outcome**	**Time to Progression (wks)**	**Status**	**Time to Death (wks)**	**Clinical Follow-up (wks)**
1	Adenoid Cystic Carcinoma	n/a	EBRT	Progressed	230	Alive	n/a	230

2	Squamous Cell Carcinoma	n/a	n/a	Regressed	n/a	Alive	n/a	192

3	Adenoid Cystic Carcinoma	n/a	n/a	Stable	n/a	Alive	n/a	161

4	Squamouc Cell Carcinoma	Resection	EBRT	Stable	n/a	Dead	142	142

5	Renal Cell Carcinoma	n/a	n/a	Stable	n/a	Alive	n/a	86

6	Adenoid Cystic Carcinoma	n/a	n/a	Stable	n/a	Alive	n/a	82

7	Renal Cell Carcinoma	n/a	n/a	Progressed	31	Alive	n/a	79

8	Melanoma	n/a	n/a	Progressed	40	Dead	77	77

9	Hemangiopericytoma	Resection	n/a	Regressed	n/a	Alive	n/a	66

10	Chondrosarcoma	n/a	n/a	Stable	n/a	Alive	n/a	52

11	Squamous Cell Carcinoma	Resection	n/a	Progressed	5	Dead	52	52

12	Rhabdomyosarcoma	n/a	n/a	Stable	n/a	Alive	n/a	49

13	Spindle Cell Carcinoma	Resection	n/a	Progressed	32	Dead	46	46

14	Transitional Cell Carcinoma	Biopsy	EBRT	Stable	n/a	Dead	41	41

15	Melanoma	n/a	n/a	Stable	n/a	Dead	39	39

16	Squamous Cell Carcinoma	n/a	EBRT	Regressed	n/a	Dead	37	37

17	Rhabdomyosarcoma	n/a	EBRT	Stable	n/a	Dead	35	35

18	Papillary Thyroid Carcinoma	n/a	n/a	Regressed	n/a	Dead	29	29

19	Leiomyosarcoma	n/a	n/a	Stable	n/a	Dead	28	28

20	Melanoma	n/a	RS	Progressed	16	Dead	21	21

21	Ewing Sarcoma	n/a	EBRT	Stable	n/a	Dead	20	20

22	Adenocarcinoma (Parotid Gland)	n/a	EBRT	Stable	n/a	Dead	18	18

23	Squamous Cell Carcinoma	n/a	n/a	Progressed	12	Alive	n/a	18

24	Hepatocellular Carcinoma	n/a	n/a	Stable	n/a	Dead	13	13

25	Squamous Cell Carcinoma	n/a	n/a	Progressed	9	Dead	13	13

26	Adenoic Cystic Carcinoma	Resection	EBRT	Regressed	n/a	Dead	11	11

27	Renal Cell Carcinoma	n/a	n/a	Stable	n/a	Dead	10	10

28	Ewing Sarcoma	n/a	EBRT	Stable	n/a	Dead	8	8

29	Adenocarcinoma (Parotid Gland)	n/a	n/a	Stable	n/a	Dead	8	8

30	Breast Carcinoma	n/a	n/a	Stable	n/a	Dead	7	7

31	Adenoid Cystic Carcinoma	Resection	EBRT	Stable	n/a	Dead	6	6

For those patients with local progression, the median time to progression was 24 weeks (range: 5 – 230 weeks). One patient with a renal cell carcinoma metastasis to the right jugular foramen/CPA who experienced local progression at 31 weeks underwent a second course of CKS, which halted further progression and resulted in subsequent local control at a follow-up of 72 weeks.

### Survival

**Figure 3:**
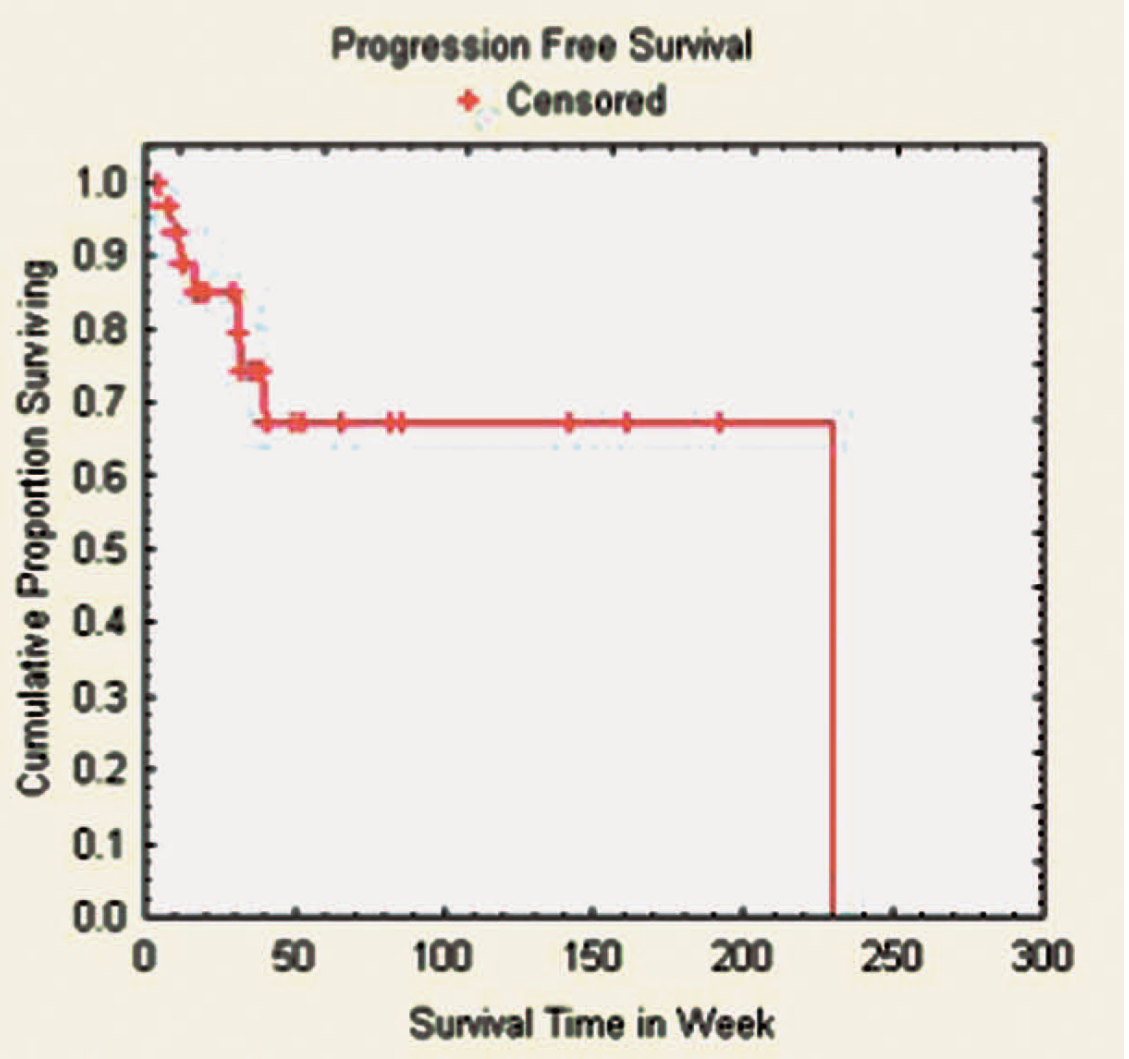
**Progression-free survival**.

**Figure 4:**
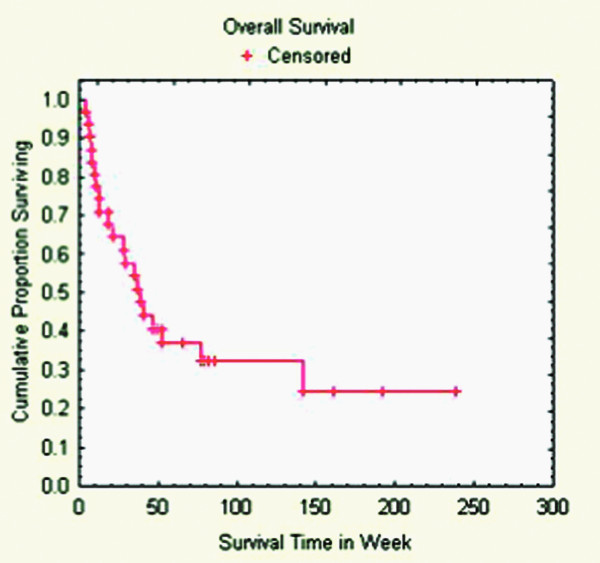
**Overall survival**.

Ten patients (32%) were alive at the end of the follow-up period, having survived a median of 81 weeks (range: 18 – 238 weeks). For the 21 patients (68%) who died, the median time to death was 25 weeks (range: 6 – 142 weeks) (Tables 4 &5). Among those patients who died, 5 (25%) had local progression. However, no patients died specifically from radiosurgery-treated disease or treatment-related complications. The median progression-free survival of the cohort was 230 weeks (Figure 3). The median overall survival of the cohort was 39 weeks (Figure 4).

### Tumor Control and Survival as a Function of "Stand-Alone" Radiosurgery versus "Adjunctive" Radiosurgery

The follow-up clinical data were compared between the groups of patients for whom CKS was primary "stand-alone" treatment versus secondary treatment following surgery or external beam radiotherapy. Among the patients with adequate follow-up data, 18 patients were treated with CKS as a primary treatment. The median follow-up was 44 weeks (range: 7 – 238 weeks). Nine patients (50%) were alive at the end of the follow-up period, and 5 (27%) experienced local tumor progression, with a median time to progression of 31 weeks (range: 9 – 230 weeks).

For the 13 patients with previous treatments for their skull base lesion, the median follow-up was 35 weeks (range: 6 – 142 weeks). One patient (8%) was alive at the end of the follow-up period, and 3 (23%) experienced local tumor progression, with a median time to progression of 16 weeks (range: 5 – 32 weeks).

### Toxicity

**Figure 5:**
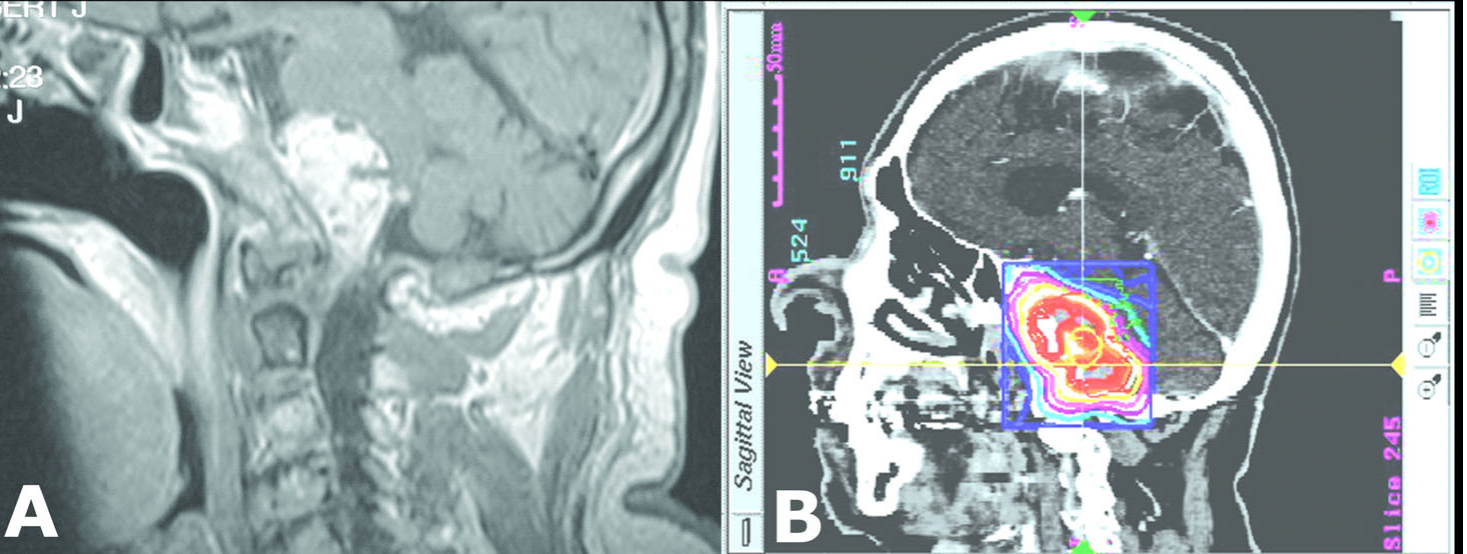
**72 year-old man with a history of transitional cell carcinoma with a biopsy proven metastasis to the clivus and foramen magnum**. He underwent prior radiation treatment with 60 Gy in 30 fractions. He presented to our institution with progressive facial numbness and difficulty swallowing. (A) Sagittal MRI of the brain after gadolinium administration demonstrating a large clival-based lesion compressing the pons and medulla. Having seen three other skull-base surgeons, none of whom offered surgical resection, we deemed the patient a good radiosurgery candidate. (B) Sagittal CT with treatment contour. The lesion was treated with 2000 cGy in 5 stages. He was followed for 41 weeks when he died of failure to thrive. There was no radiographic progression of this lesion at the time of his last follow-up appointment.

The neurological deficits before and after CKS are summarized in Table 6. Altered vision comprised the most common presenting symptom prior to radiosurgery, with 10 patients having reduced visual acuity, 13 patients having diplopia, and 1 patient having proptosis. Four patients (40%) experienced improved visual acuity and three patients (23%) experienced improvement from their diplopia following treatment. Otherwise, all symptoms remained stable at last follow-up. Of the 17 patients with facial weakness or facial pain on physical examination prior to CKS, 15 (88%) remained stable at last follow-up. One patient (6%) with facial weakness reported improvement. In one patient, facial weakness and swallowing difficulty worsened following CKS due to local disease progression involving all cranial nerves. Swallowing difficulties were found in four patients, 75% of which remained stable following treatment (Figure 5). In the absence of tumor progression, there were no cranial nerve, brainstem or vascular complications referable specifically to CyberKnife^® ^radiosurgery. Specifically, there were no new cranial nerve deficits observed following SRS in this series.

**Table 6:** Summary of neurological deficits before and after CyberKnife radiosurgery

	**No. of Patients**			
	
**Deficit**		**Post-CKS**		
		
	***Pre-CKS***	Improved	Stable	Worse
Reduced visual acuity	10	4	6	0
Diplopia	13	3	10	0
Proptosis	1	0	1	0
Facial weakness	10	1	8	1
Facial pain	7	0	6	1
Swalowing difficulty	4	0	3	1
Hearing loss	3	0	3	0

## Discussion

Skull base malignancies pose unique challenges to the clinician because of oncological and neurological factors. Since these tumors present late in the course of the patients' disease, they are often poor candidates for aggressive therapy. And because these tumors are in close proximity or contact with the brain stem and cranial nerves, complete surgical resection is almost uniformly impossible without significant neurological injury. External beam radiation has had limited success in treating these malignancies largely due to dose-limitations [27,28]. Given the results of the current study, we feel that microsurgical resection of skull base malignancies may no longer be the "gold-standard" or optimal first-line treatment. Cases should be evaluated on an individual basis by a multi-disciplinary team so that the best treatment, capitalizing on the advances in skull base microsurgery and radiation oncology, can be delivered.

### Review of the Literature

Radiosurgery may be uniquely suitable for treating these tumors, since it is non-invasive and can precisely target the tumor with minimal spread of radiation to surrounding normal neurological structures. Various investigators have reported their experience with stereotactic radiosurgery in the treatment of malignant skull base tumors. Cmelak et al. reported their data on 47 patients with 59 malignant skull base tumors [54]. Eleven patients with primary nasopharyngeal carcinoma were treated with Linac radiosurgery as a boost (7 – 16 Gy, median: 12 Gy) after a course of fractionated radiotherapy. None of the eleven had tumor progression during the follow-up period. The rest of the patients were treated for skull base metastases or local recurrences from primary head and neck cancers. Radiation doses of 7.0 Gy – 35.0 Gy (median 20.0 Gy) were delivered to these lesions, usually as a single fraction. A tumor control rate of 69% was reported for these patients during the study period (median: 36 weeks). Major toxicities occurred after 5 of 59 treatments. These included three cranial nerve palsies, one CSF leak, and one case of trismus. An important conclusion from their data was that local control did not correlate with lesion size, histology, or radiosurgical dose.

Two small studies from Japan showed similar results. Tanaka et al. reported on 19 malignant skull base tumors, which they treated with single fraction gamma knife radiosurgery [33]. The mean marginal dose utilized was 12.9 Gy. During a follow-up period of 22 months, a tumor control rate of 68% was recorded. The other study by Iwai and Yamanaka of 18 similar patients showed a tumor control rate of 67% during a median follow up of 10 months [31]. A local control rate as high as 95% at 2 years has been reported in one radiosurgery study, but the patient population in that series included 66% with skull base chordomas, chondrosarcomas and adenoid cystic carcinomas, which differ significantly from the cancer patient population studied in the other cited series and our own [55].

In the attempt to bring some order to a heterogenous group of skull base tumors, Morita et al. recently classified cranial base tumors by the degree of aggressiveness into benign, intermediate malignant (or low grade/slow growing), and highly malignant (or fast growing) [56]. Applying this strategy to our series, 31 tumors in our series (84%) would be classified as "highly malignant" or fast growing. Despite this unfavorable bias in our population, the tumor control rate in our series compared favorably to the rate reported in the literature [3,31,33,54,57]. We treated 31 malignant skull base tumors with a median marginal dose of 2500 cGy delivered in 2–7 sessions (median of 5) and achieved a local control rate of 74% during the follow-up period (median 37 weeks). The median progression-free survival was 230 weeks. In separate analysis of the patients with tumors classified as "highly malignant", the local control rate in this sub-group of patients did not differ significantly from the total study population (74% at 40 weeks), confirming the reported finding on metastatic tumors that response to radiosurgery may be independent of tumor characteristics [15]. Similarly, a comparison of patients who received radiosurgery as primary treatment versus adjunct treatment after surgery or radiotherapy did not reveal major differences in outcome.

### Limitation of Toxicity

Neurological deterioration occurred only in a minority of our patients and in each case, it was accompanied by local tumor progression. Neurological symptoms remained stable or improved in 94% of the patients. No neurological deficits were attributable to toxicity of radiosurgery. Although it is possible that a higher complication rate will emerge with longer follow-up, we believe that the lack of morbidity is largely the result of delivering radiosurgery in multiple sessions, with high conformality and homogeneity. Fractionation is a cornerstone principle in radiation oncology. The oncologist uses it to exploit the significantly different response to radiation of normal versus neoplastic tissue, for the protection of the former and ablation of the latter. It provides time for normal tissue repair between doses, and theoretically minimizes radiation toxicity. With the advent of frameless, image-guided radiosurgery, "hypofractionation" or multi-session treatment became possible. Adler et al. reported on their experience on multi-session radiosurgery for treating skull base, benign tumors situated within 2 mm of the optic apparatus. They achieved a high tumor control rate and found that 94% of the patients had stable or improved vision after treatment [46]. The authors believed that staging the treatment significantly contributed to the low incidence of radiosurgical toxicity. In addition to protective effects, the staging of radiosurgical treatments may have heretofore under-recognized tumor control benefits as well. A new report from Canada showed that patients who received staged radiosurgery to their brain metastases survived longer that those who received single-session treatment [58]. It is possible, that by allowing for a higher total dose delivery to the tumor, staging may lead to better tumor control.

A recent report out of our institution demonstrated that the CyberKnife^® ^radiosurgical system is capable of delivering a high dose of radiation to a well-defined clinical target volume with high conformity (median NCI 1.66) and homogeneity (median HI 1.26), regardless of irregular tumor shape, large tumor volume, or proximity to critical structures [59]. The median NCI in the present series was 1.60, and the median HI was 1.32. Although still controversial, it is our opinion that improved conformity and homogeneity may maintain high rates of local control while decreasing radiation-induced complications [53,59-61]. It seems intuitively evident that conformality and homogeneity are important in treating malignancies of the skull base, since all the tumors are in close proximity to, or entirely surround critical neurological structures that have limited radiation tolerance. In many instances, the encircled cranial nerve is not visible on the treatment-planning image, and one must assume that it received the maximum dose.

### Dose and Staging Selection

A significant majority of the patients in the present study received a does of 2500 cGy in 5 stages. The initial selection of the dose and staging regimen stemmed from our group's experience using the CyberKnife^® ^radiosurgical system to treat benign skull base lesions. Having encountered no neurological morbidity attributable to radiosurgery in this study, it is impossible to tell whether current treatment regimen represent the "ideal" dose to malignant skull base tumors. A higher average dose may lead to a better tumor control rate than the 74% seen in the present series, and still achieve an acceptably low rate of complications. It is also possible that the "ideal" dosing and staging is different for each patient, dependent on histopathology, previous treatments, tumor volume, neurological status and systemic tumor burden. Our confidence in raising the treatment dose, like the "true" complication rate, will no doubt come with time and further experience with these difficult tumors.

## Conclusion

Despite the significant challenges, stereotactic radiosurgery appears to be a safe and reasonably effective treatment modality for the treatment of malignant primary, recurrent, and metastatic skull base tumors. Our experience suggests that image-guided, multi-session radiosurgery compares favorably to other radiosurgical techniques in the treatment of these difficult tumors. In addition, no major morbidity was observed as a direct result of this method. Longer follow-up and, optimally, comparison of dosimetry and other treatment parameters across institutions, will be necessary to more accurately define the long-term survival and effect of multi-session radiosurgery on disease progression for patients with these aggressive tumors.

## Competing interests

The authors declare that they have no competing interests.

## Authors' contributions

NC performed the chart review, organized data, analyzed data, drafted manuscript, created tables, obtained images. DR assisted in the chart review, organization of data, and drafting of manuscript. YZ performed the statistical analysis and created statistical figures. BC participated in the treatment planning of patients included in this study. KH participated in the treatment planning of patients included in this study. GG participated in the treatment planning of patients included in this study. SC assisted in the organization of data, data analysis, table construction, literature review, and participated in the treatment planning of patients included in this study. WJ conceived of the study, participated in its design and coordination. Assisted in data analysis and drafting of manuscript.
